# Small Heterodimer Partner-Targeting Therapy Inhibits Systemic Inflammatory Responses through Mitochondrial Uncoupling Protein 2

**DOI:** 10.1371/journal.pone.0063435

**Published:** 2013-05-21

**Authors:** Chul-Su Yang, Jae-Min Yuk, Jwa-Jin Kim, Jung Hwan Hwang, Chul-Ho Lee, Jin-Man Kim, Goo Taeg Oh, Hueng-Sik Choi, Eun-Kyeong Jo

**Affiliations:** 1 Department of Microbiology, Chungnam National University School of Medicine, Daejeon, S. Korea; 2 Department of Pathology, Chungnam National University School of Medicine, Daejeon, S. Korea; 3 Infection Signaling Network Research Center, Chungnam National University School of Medicine, Daejeon, S. Korea; 4 Laboratory Animal Center, Korea Research Institute of Bioscience and Biotechnology, Daejeon, S. Korea; 5 Division of Life and Pharmaceutical Science, Ewha Womans University, Seoul, S. Korea; 6 National Creative Research Initiatives Center for Nuclear Receptor Signals, Hormone Research Center, School of Biological Sciences and Technology, Chonnam National University, Gwangju, S. Korea; Virginia Tech, United States of America

## Abstract

The orphan nuclear receptor, small heterodimer partner (SHP), appears to play a negative regulatory role in innate immune signaling. Emerging evidence warrants further study on the therapeutic targeting of SHP to suppress excessive and deleterious inflammation. Here we show that fenofibrate, which targets SHP, is required for inhibiting systemic inflammation via mitochondrial uncoupling protein 2 (UCP2). *In vivo* administration of fenofibrate ameliorated systemic inflammatory responses and increased survival upon experimental sepsis through SHP. An abundance of SHP was observed in mice fed fenofibrate and in cultured macrophages through LKB1-dependent activation of the AMP-activated protein kinase pathway. Fenofibrate significantly blocked endotoxin-triggered inflammatory signaling responses via SHP, but not via peroxisome proliferator-activated receptor (PPAR)-α. In addition to the known mechanism by which SHP modulates innate signaling, we identify a new role of fenofibrate-induced SHP on UCP2 induction, which is required for the suppression of inflammatory responses through modulation of mitochondrial ROS production. These data strongly suggest that the SHP-inducing drug fenofibrate paves the way for novel therapies for systemic inflammation by targeting SHP.

## Introduction

Nuclear receptors (NRs), a unique family of ligand-modulated transcription factors, orchestrate numerous aspects of mammalian physiology, such as lipid and glucose metabolism, reproduction, development, and homeostasis [1], [2]. In humans, 48 members of the NR superfamily are known, including NR with known ligands (retinoids or thyroid hormone) and orphan NRs with unidentified ligands [3]–[5]. Among the orphan members of the NR superfamily, “small heterodimer partner (SHP; also called NR0B2)” contains a ligand-binding domain but lacks the conserved DNA binding domain that interacts with NR, including thyroid receptor, retinoic acid receptors, and estrogen receptors α and β [4], [6]. SHP is a key transcriptional regulatory factor for a variety of genes that participate in diverse metabolic functions and pathways, including lipid and bile acid metabolism, as well as glucose homeostasis [4], [5], [7]. Although conflict remains regarding the discovery of direct SHP ligands, several pathways have been characterized that induce SHP expression [8]. Previous studies showed that SHP expression is induced by numerous hormones, molecules, and drugs, including the anti-diabetic drug metformin [9], hepatocyte growth factors [10], fenofibrate [11], and sodium arsenite [12].

Although inflammation is fundamentally beneficial for the host against pathogenic challenge or injury, prolonged or exacerbated inflammatory responses can be detrimental, resulting in pathologic responses in diverse disease setting such as local or systemic inflammation [13], [14]. Currently, accumulating evidence has revealed that several members of the NR superfamily regulate immune and inflammatory responses through specific modes of interaction, and/or regulation of gene expression, to maintain homeostasis in the body [15], [16]. SHP also seems to play a crucial role in regulation of inflammation. Generally, SHP is thought to inhibit signal-dependent activation of inflammation through transrepression via interactions with diverse co-regulatory proteins and transcription factors [4], [15]. For example, SHP can be induced in vascular smooth muscle cells and inhibits vascular inflammatory responses as a target gene of farnesoid X receptor/bile acid receptor (FXR; NR1H4) [17]. We previously showed that SHP negatively regulates toll-like receptor (TLR)-dependent inflammation through a biphasic interaction in the cytosol with the signaling molecules NF-κB and tumor necrosis factor receptor-associated factor 6 (TRAF6) [18].

Mitochondrial uncoupling protein 2 (UCP2) is one of the mitochondrial anion carrier proteins that fundamentally mediate mitochondrial proton leaks [19], [20]. UCP2 is involved in a variety of physiologic processes related to glucose and lipid metabolism, and also plays an essential role in various pathologic conditions, including obesity, diabetes, and atherosclerosis [20]. UCP2 is expressed in multiple tissues, and mainly functions in the protection against oxidative stress [19], [20]. Earlier studies reported an important role for UCP2 in minimizing mitochondrial reactive oxygen species (ROS) generation from the electron transport chain and macrophage-mediated immunity against infection [21]. UCP2-deficient mice have increased IL-1β and nitric oxide production, and stronger inflammatory responses in islets, leading to the development of autoimmune diabetes [22]. Although UCP2 modulates macrophage regulation of inflammatory function, how UCP2 gene and protein expression are regulated remains unidentified.

A major question is whether SHP inducing agents or drugs may play a role in the inhibition of systemic excessive inflammation. In this study, to induce SHP expression, we used fenofibrate, a drug that reduces cholesterol and triglyceride levels. We clearly demonstrate that therapeutic administration of fenofibrate ameliorated systemic inflammatory responses and increased survival of experimental sepsis through SHP. Fenofibrate-dependent inhibition of pro-inflammatory cytokine production was dependent on SHP, but not peroxisome proliferator-activated receptor (PPAR)-α. Importantly, SHP-mediated UCP2 expression was required for the fenofibrate-mediated inhibition of pro-inflammatory responses through modulation of mitochondrial ROS generation. Our findings indicate, therefore, that targeting SHP may represent a novel strategy to ameliorate excessive inflammatory responses.

## Materials and Methods

### Mice and Sepsis Model

Wild-type C57BL/6 mice were purchased from KOATECH (Pyungtek, Korea), and mice with a targeted deletion in the SHP gene (homozygous mice and their homozygous littermates) were generated as previously described [23]. PPARα^−/−^ mice were kindly provided by Dr. Goo Taeg Oh (Ewha Womans University, Seoul, Korea). Male and female mice were used at 6–8 weeks of age. All animal-related procedures were reviewed and approved by the Institutional Animal Care and Use Committee, Chungnam National University School of Medicine (Daejeon, Korea). The mice used for the LPS challenge were 8–10 weeks old. Fenofibrate was diluted in corn oil and orally administered for 3 or 7 consecutive days. The experimental groups included age- and sex-matched animals. *Escherichia coli* O26:B6 LPS (Sigma) was diluted in sterile PBS and injected into the animals intraperitoneally (i.p.), as described previously [24]. Viability was monitored every 12 h for 7 days. There was no further increase in death after 96 h. Mice were monitored daily for weight loss or other signs of morbidity (activity, eating, posture, and coat condition) over a 7 days period. Moribund mice or mice losing more than 25% body weight were sacrificed by CO_2_ asphyxiation, and cardiac puncture was performed to collect blood.

### Cell Culture

Primary bone marrow-derived macrophages (BMDMs) were differentiated for 5–7 days in M-CSF–containing media, as described previously [18]. The culture media consisted of DMEM supplemented with 10% heat-inactivated FBS, 1 mM sodium pyruvate, 50 U/ml penicillin, 50 µg/ml streptomycin, and 5×10^−5^ M 2-mercaptoethanol. The mouse macrophage cell line RAW264.7 (ATCC TIB-71; American Type Culture Collection) was maintained in DMEM/glutamax supplemented with 10% FBS.

### Reagents

For *in vitro* experiments, LPS was purchased from Invitrogen. Fenofibrate and Compound C were from Sigma. Dimethyl sulfoxide (DMSO; Sigma) was added to the cultures at 0.1% (v/v) as a solvent control. Specific antibodies (Abs) against phospho-Acetyl-CoA Carboxylase (3661), phospho-AMPKα (2535), LKB1 (3047), and phosphor-IκBα (9246) were purchased from Cell Signaling. Anti-TRAF6 (sc-7221 and sc-8409), anti-Ub (sc-8017), anti-IκBα (sc-371), anti-NFκB p65 (sc-372), anti-SHP (sc-30169 and sc-15283), anti-UCP2 (sc-6525), anti-HMG-1 (sc-74085), anti-PPARα (sc-9000), anti-CaMKKβ (sc-9630), and anti-Actin (sc-1616) were purchased from Santa Cruz Biotechnology. Anti-COX IV (ab16056) was purchased from Abcam. All other reagents were purchased from Sigma.

### Cellular Fractionation

After cell stimulation was terminated by the addition of ice-cold PBS, nuclear and cytosolic protein extracts were prepared using the Mitochondrial Fractionation Kit (Active Motif) according to the manufacturer’s instructions. All steps of subcellular fractionation were performed at 4°C. Fraction purity was evaluated by western blotting using actin as a cytoplasmic marker and COX IV as a mitochondrial marker.

### Adenovirus Production

Adenoviruses (Ad) encoding GFP only (Ad-GFP), Ad-DN-AMPK, Ad-CA-AMPK, full-length human SHP (Ad-SHP), and Ad-siSHP were prepared, as described previously [18]. Large-scale amplification of adenovirus and viral titers was performed as previously described [24]. In brief, HEK293A cells were transduced with adenoviral vector (multiplicity of infection = 2), and the replicated virus particles were concentrated using CsCl gradient ultracentrifugation. Purified and concentrated adenoviruses that had titers in the range of 10^9^–10^11^ PFU/ml were suspended in 10 mM Tris-HCl (pH 8.0), 2 mM MgCl_2_, and 5% sucrose. For *in vitro* infection with adenovirus, the BMDMs were plated at 5×10^5^ cells per well in 48- well tissue culture plates in DMEM plus 2% FBS that contained recombinant adenovirus at a concentration of 10 PFU per cell, according to the method described previously [18].

### Lentiviral shRNA Production

For silencing murine CaMKKβ, LKB1, or UCP2 in primary cells, pLKO.1-based lentiviral CaMKKβ shRNA constructs (sc-38952-SH) and LKB1 shRNA constructs (sc-35817-SH) were obtained from Santa Cruz Biotechnology. UCP-2 shRNA constructs (RMM4534-NM_011671) were purchased from Open Biosystems. Lentiviruses were produced by transient transfection using packaging plasmids (pMDLg/pRRE, pRSV-Rev, and pMD2.VSV-G, purchased from Addgene) after Lipofectamine 2000-mediated transient transfection into HEK293T cells, as described previously. Virus-containing media was collected at 72 h post-transfection and concentrated by ultracentifugation. Lentiviral vector titration was determined using 293T cells and the generated lentiviruses were transduced into BMDMs, as described previously [18].

### *In vivo* Lentivirus Transduction

Concentrated lentiviral particles were thawed at 4°C and diluted in PBS and polybrene (8 µg/mL final concentration; Sigma) to give a dose of 1×10^9^ pfu in a 100 µl injection volume, as described previously [24]. Mice were injected with lentivirus (administered intravenously) expressing nonspecific shRNA (shNS) or shRNA specific for PPARα (shPPARα) or UCP2 (sh-UCP2) for 2 consecutive days and then orally administered fenofibrate for 7 consecutive days before LPS challenge. Perform desired experiments.

### RNA Extraction, Semi-quantitative RT-PCR, Western Blotting, and ELISA

RNA extraction and semi-quantitative RT-PCR were performed as described previously [24]. The sequences of the primers used were as follows: mTNF-α (forward: 5′-CGGACTCCGCAAAGTCTAAG-3′, reverse: 5′-ACGGCATGGATCTCAAAGAC-3′), mIL-6 (forward: 5′-GGAAATTGGGGTAGGAAGGA-3′, reverse: 5′-CCGGAGAGGA-GACTTCACA G-3′), mIL-1β (forward: 5′-CTCCATGAGCTTTGTACAAGG -3′, reverse: 5′-TGCTGATGTAC CAGTTGG GG-3′), mIL-10 (forward: 5′-ATGCAGGACT-TTAAGGGTTA-3′, reverse: 5′-ATTTCGGAGAGAGGTACAAA-3′), mSHP (forward: 5′-CTCTGCAGGTCGTCCGACTATTC TG-3′, reverse: 5′-CCTCGAAGGTCACAGCAT-CCTG-3′), mUCP2 (forward: 5′-CTACAAGA CCATTGCACGAGAGG-3′, reverse: 5′-AGCTGCTCATAGGTGACAAACAT-3′), mUCP3 (forward: 5′-GGAGCCATGGCAGTGACCTGT-3′, reverse: 5′-TGTGATGTTGGGCCAAGTC CC-3′), mβ-actin (forward: 5′-TCATGAA GTGTGACGTTGACATCCGT-3′, reverse: 5′-CCT AGAAGCATTTGCGGTGCACGATG-3′).

For western blot analyses, primary antibodies were used at 1∶1000 dilutions. The membranes were developed using chemiluminescence assays (ECL; Pharmacia-Amersham) and subsequently exposed to chemiluminescence film (Pharmacia-Amersham). In the sandwich ELISA, serum and cell culture supernatants were analyzed using DuoSet antibody pairs (Pharmingen, San Diego, CA, USA) for the detection of interleukin (IL)-6, IL-1β, IL-10, and TNF-α.

### Detection of HMGB1

For detect HMGB1 in cell culture supernatants and sera, samples containing equal amounts of proteins were a trichloroacetic acid (TCA) precipitated. Experiments were performed at 4°C, as previously described [25]. In brief, one volume of 20% TCA was mixed with one volume of protein sample, and the mixture was vortexed. After 1 h of incubation at −20°C, the sample was centrifuged at 15,000×*g* for 15 min at 4°C, and the supernatant was removed. Then, 0.5 mL of ice-cold acetone containing 20 mM DTT was added, and the mixture was centrifuged at 13,000×*g* for 15 min at 4°C. The supernatant was discarded, and the pellet was air dried. The samples were subjected to IB with anti-HMGB1 in cell culture supernatants and sera or whole cell lysate.

### Immunoprecipitation and *in vivo* Ubiquitination Assays

Immunoprecipitation and *in vivo* ubiquitination assays were performed as described [18]. For immunopreciptiation, cells were rinsed twice with ice-cold PBS, and solubilized for 30 min on ice in NP-40 lysis buffer [20 mM Tris (pH 7.5), 150 mM NaCl, 2 mM EDTA, 10% glycerol, 1% NP-40] containing complete protease and phosphatase inhibitor cocktail (Roche Applied Science). Lysates were centrifuged at 15,000 g at 4°C for 15 min. Proteins (500 mg) were immunoprecipitated for 18 h with the indicated antibodies (1 µg). Prewashed Protein G sepharose beads (GE Healthcare) were added to each sample, followed by incubation for an additional 4 h at 4°C and four washes in lysis buffer. The samples were separated by SDS–PAGE, transferred to a PVDF membrane, and analyzed by western blotting.

For detection of *in vivo* ubiquitination of TRAF6, SDS was added to lysates at a final concentration of 1%. Lysates were boiled for 10 min to remove noncovalently attached proteins, followed by immunoprecipitation with anti-TRAF6. Samples were subsequently solubilized in SDS sample buffer [80 mM Tris-HCl (pH 6.8), 2% SDS, 50% glycerol, 0.05% bromophenol blue, 0.2 M DTT] for immunoblot analyses with either anti-ubiquitin or anti-TRAF6 antibodies.

### Confocal Fluorescence Microscopy

Translocation of NF-κB p65 into the nucleus was detected using immunofluorescence staining as previously described [18]. Cells were fixed for 15 min in 4% paraformaldehyde in PBS, permeabilized for 15 min with 0.25% Triton X-100 in PBS, and treated with 1% BSA for 1 h at 25°C. The cells were incubated overnight at 4°C with a diluted (1∶400) mouse antibody against NF-κB p65. After washing to remove excess primary antibody, cells were incubated for 1 h at room temperature with a fluorophore-conjugated secondary antibody (anti-mouse IgG-Cy2 or anti-mouse IgG-PE Ab). Excess antibody was removed, and cells were imaged using a confocal microscope (LSM510 META; Carl Zeiss). In some experiments, cells were stained with DAPI (Sigma) to visualize the nuclei, and washed three times with PBS before imaging. The fluorescence intensity of each field was measured using ImageJ analysis software or Adobe Photoshop CS4™ software. Analyses of co-localization data were based on the pixel histogram using the ImageJ analysis software or Adobe Photoshop CS4™ software. In all experiments, a minimum of 150 cells per sample were counted and samples in duplicate or triplicate were counted per experimental condition.

### Immunohistostaining

For immunohistostaining of tissue sections, spleens were fixed in 10% formalin and sectioned in paraffin, as previously described [18]. To examine TNF-α, COX-2 or iNOS expression, 3-µm paraffin sections were deparaffinized and hydrated by serially dipping into 100–70% ethanol, distilled water, and PBS. The slides were antigen retrieved in sodium citrate buffer and blocked for 20 min in 1.5% normal rabbit serum in PBS and stained for TNF-α (sc-52746), COX-2 (sc-7951) or iNOS (sc-8310, Santa Cruz).

### Statistical Analyses

For statistical analyses, data obtained from independent experiments were analyzed using a paired *t*-test with Bonferroni adjustment and are presented as the mean ± SD. Differences were considered significant at *p*<0.05. Where indicated, GraphPad Prism (GraphPad Software, Inc.) was used for two-way analysis of variance and Kaplan-Meier survival analyses.

## Results

### Fenofibrate Suppresses LPS-induced Lethal Systemic Inflammatory Responses via Induction of SHP *in vivo*

**Figure 1:**
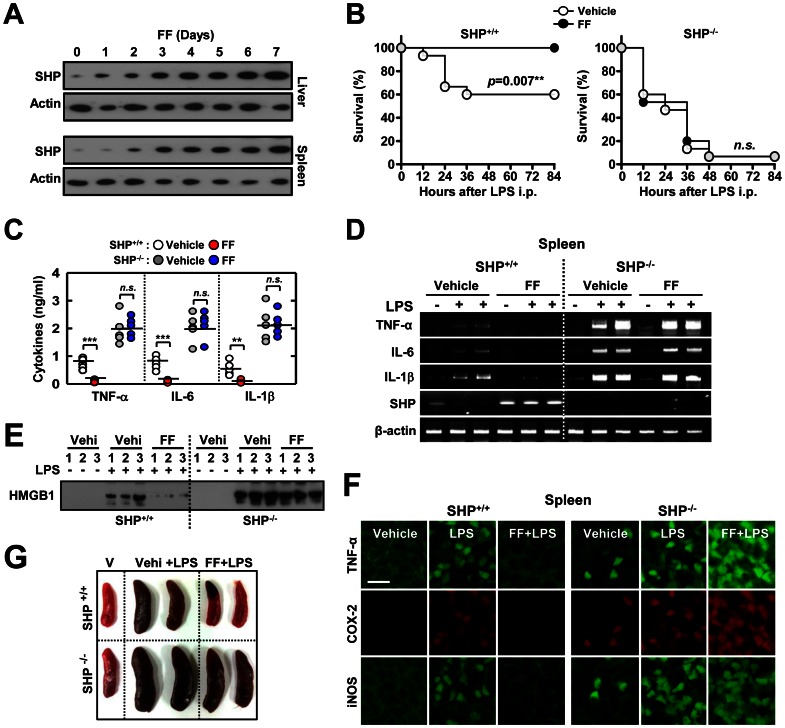
Fenofibrate protects mice from LPS-induced lethal shock and systemic inflammation through SHP. *Shp* ^+/+^ and *Shp* ^−/−^ mice received either fenofibrate (100 mg/kg; administrated orally) or vehicle for the indicated period (**A**) or 7 consecutive days (**B** to **G**) before LPS injection (30 mg/kg; *n* = 15 each group, i.p). (**A**) SHP expression in liver and spleen was assessed by immunoblotting (IB). Whole cell lysates (WCL) were used for IB with anti-Actin. (**B**) The survival of *Shp* ^+/+^ and *Shp* ^−/−^ mice was monitored for 84 h. Results are presented as the mean ± SEM. *n.s*., non-specific (log-rank test)**.** (**C**) Sera were collected from *Shp* ^+/+^ and *Shp* ^−/−^ mice (n = 5 each group) at 18 h post-LPS injection and the concentrations of TNF-α, IL-6, and IL-1β were measured by ELISA. (**D**) Expression of *Tnfα*, *Il6*, *Il1b,* and *Shp* mRNA in spleen tissues was analyzed using RT-PCR at 6 h after i.p injection of LPS. (**E**) Sera were collected from mice at 24 h post-LPS injection and subjected to IB to detect HMGB1 expression. (**F**) Representative immunofluorescence images for expression of TNF-α, COX-2, and iNOS in spleen tissues from *Shp* ^+/+^ and *Shp* ^−/−^ mice. Scale bar, 50 µm. (**G**) Spleen size was measured in the presence of vehicle only (V; *each left*), vehicle plus LPS (Vehi+LPS; *each middle*), and fenofibrate plus LPS (FF+LPS; *each right*)-injected *Shp* ^+/+^ (top) and *Shp* ^−/−^ (bottom) mice (at 18 h). The data (**A**, **D**, **E**, **G**, and **F**) are representative of at least three independent experiments with similar results. Quantitative data are shown as the mean ± SD of three experiments (**C**). Statistical differences (***, *p*<0.001), as compared to the control mice, are indicated (paired *t*-test with Bonferroni adjustment). FF, fenofibrate.

In this study, we evaluated the contribution of fenofibrate in the induction of SHP in various tissues and, particularly, hematopoietic cells. We determined whether fenofibrate-induced SHP is required for inhibiting systemic inflammation *in vivo*. Our previous study identified that *in vitro* induction of SHP by macrophage stimulating protein regulates TLR-dependent inflammatory responses [18]. However, it has not been investigated whether SHP-inducing drugs could suppress systemic inflammation *in vivo*. We confirmed that SHP is induced in liver, spleens, and bone marrow in mice after oral administration of fenofibrate at day 3 (Fig. 1A and data not shown).

To determine the *in vivo* protective effects of fenofibrate in systemic inflammation, wild-type (WT) and SHP knockout (KO) mice were subjected to a model of endotoxin-induced septic shock by intraperitoneal injection with LPS [18]. Most deaths in the WT group occurred within 36 h after injection, whereas the fenofibrate-treated group did not have any observed deaths until 84 h (Fig. 1B). However, septic shock was dramatically accelerated and exacerbated in SHP KO mice treated with or without fenofibrate, who succumbed to the same dose of LPS as WT mice (Fig. 1B). Consistent with the survival rate, the expression of proinflammatory cytokines from sera (Fig. 1C) and different tissues [Fig. 1D (spleen) and data not shown] were significantly decreased in fenofibrate-treated WT mice, whereas they were markedly elevated in SHP KO mice upon treatment with fenofibrate (Fig. 1C and 1D). Moreover, the increased serum levels of HMGB1 (Fig. 1E), the expression of tumor necrosis factor (TNF)-α, cyclooxygenase-2, and inducible nitric oxide synthase in spleens (iNOS; Fig. 1F) were significantly attenuated in LPS-challenged WT mice upon treatment with fenofibrate, whereas this was not observed in SHP-deficient mice (Fig. 1E and F). We further examined the pulmonary histology between WT and SHP KO mice, and observed higher alveolar neutrophil infiltration after LPS challenge in SHP KO mice, as compared to WT mice (data not shown). Although fenofibrate treatment significantly inhibited neutrophil infiltration in WT mice, this was not observed in SHP KO mice (data not shown). Gross examination also showed that many SHP KO mice had splenomegaly after LPS challenge, which remained largely unchanged with fenofibrate treatment (Fig. 1G). Thus, these results indicate that *in vivo* administration of fenofibrate contributes to ameliorating systemic inflammation and septic shock through SHP expression.

### Fenofibrate Suppresses TLR-dependent Inflammatory Responses via SHP

**Figure 2:**
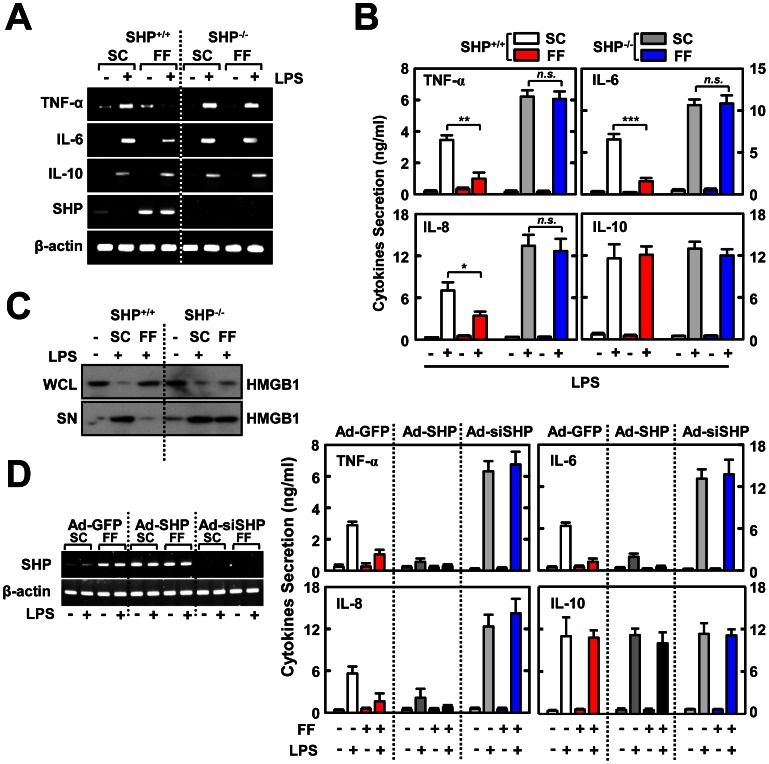
Fenofibrate inhibits LPS-mediated pro-inflammatory cytokine generation by induction of SHP. BMDMs from *Shp* ^+/+^ and *Shp* ^−/−^ mice were pretreated with fenofibrate (50 µM) for 4 h and then stimulated with LPS (100 ng/mL) for 6 h (**A**), 18 h (**B**), or 48 h (**C**). (**A**) Semi-quantitative RT-PCR analyses of *Tnfα*, *Il6*, *Il10*, *shp,* and *Actb* mRNA expression. (**B**) ELISA analyses of TNF-α, IL-6, IL-8, and IL-10 proteins in culture supernatants. (**C**) Immunoblot analysis of HMGB1 protein in whole cell lysate (WCL) and supernatant (SN). (**D**) ELISA analyses of TNF-α, IL-6, IL-8, and IL-10 proteins in culture supernatants. BMDMs from WT mice were transduced with Ad-GFP, Ad-SHP, or Ad-siSHP (MOI = 10) for 48 h, followed by treatment with fenofibrate (50 µM) for 4 h prior to LPS (100 ng/mL) stimulation. *Left*, Semi-quantitative RT-PCR analyses of the efficiency of adenoviral transduction. The data (**A**, **C**, and **D** *left*) are representative of at least three independent experiments with similar results. Quantitative data are shown as the mean ± SD of three experiments (**B**, and **D** *right*). Statistical differences (**, *p*<0.01; ***, *p*<0.001), as compared to the control mice, are indicated (paired *t*-test with Bonferroni adjustment). FF, fenofibrate; *n.s.*, non-specific.

To determine whether fenofibrate-induced SHP is required for the inhibition of TLR-induced inflammatory signaling, BMDMs from WT and SHP KO mice were cultured with or without fenofibrate, and the expression of TNF-α, IL-6, IL-8, and IL-10 were evaluated (Fig. 2A). LPS-induced *TNF-α* and *IL-6*, but not *IL-10*, were significantly decreased in fenofibrate-treated BMDMs from WT mice. These effects were not observed in cells from SHP KO mice (Fig. 2A). A similar reduction was observed in inflammatory cytokine synthesis (Fig. 2B) and HMGB1 secretion (Fig. 2C) in fenofibrate-treated WT BMDMs, but not in SHP-deficient BMDMs. We next sought to determine whether overexpression or knockdown of SHP affected the fenofibrate-induced regulation of inflammatory cytokine production. As shown in figure 2D, SHP overexpression led to a significant decrease in cytokine synthesis, whereas SHP knockdown resulted in a marked increase in cytokine synthesis, with or without fenofibrate treatment. These results demonstrate that fenofibrate regulates LPS-mediated inflammatory responses through SHP.

### Fenofibrate-induced SHP Suppresses TLR4-dependent NF-κB Signaling via its Interaction with NF-κB p65 and Inhibition of TRAF6 Polyubiquitination

**Figure 3:**
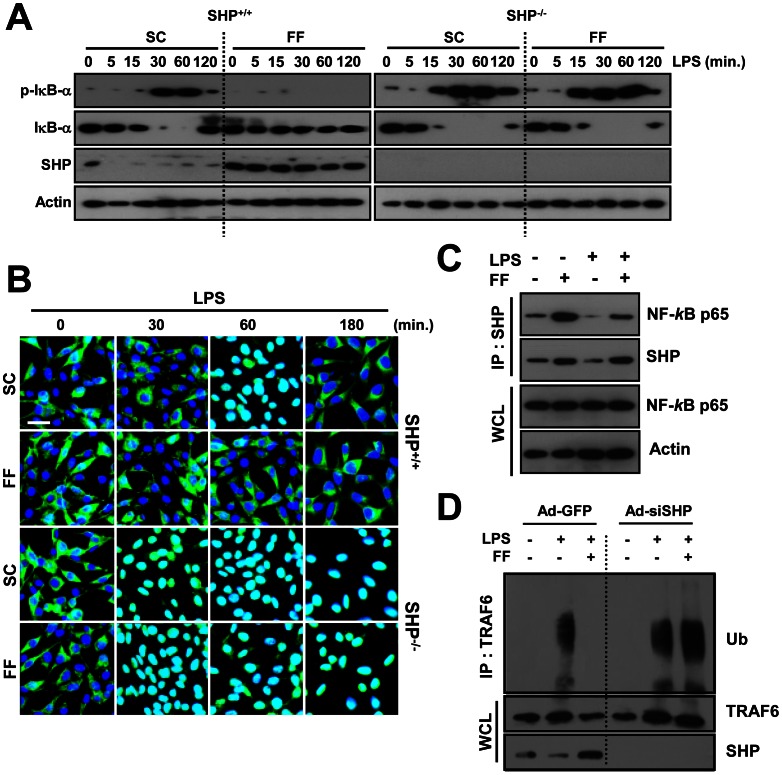
Fenofibrate-induced SHP regulates TLR4 signaling through interaction with NF-κB p65 and modulation of TRAF6 ubiquitination. (**A, B,** and **D**) BMDMs from *Shp* ^+/+^ and *Shp* ^−/−^ mice were treated with or without fenofibrate (50 µM) for 4 h, and then stimulated with LPS (100 ng/mL) for indicated times. (**A**) Immunoblotting (IB) was performed to determine protein expression of phosphorylated and total forms of IκB-α and SHP. Actin was used as a loading control. (**B**) Immunofluorescence analyses of NF-κB p65 nuclear translocation. Cells were fixed and stained with anti-NF-κB p65 (green); nuclei were counterstained with DAPI (blue). Scale bar, 20 µm. (**C**) RAW264.7 cells were treated with or without fenofibrate (50 µM) for 4 h, followed by LPS stimulation (100 ng/mL, 30 min). Cells were then subjected to immunoprecipitation (IP) with anti-SHP. Protein interactions between SHP and NF-κB p65 were analyzed by IB with anti-NF-κB p65. Whole cell lysates (WCL; input control) were detected by immunoblotting with anti-NF-κB p65 and anti-Actin. (**D**) Cell lysates were subjected to IP with anti-TRAF6 and the polyubiquitination of TRAF6 was analyzed by IB with anti-ubiquitin (Ub). WCL were detected by IB with anti-TRAF6 or anti-SHP as loading controls. The data are representative of at least three independent experiments with similar results. SC, solvent control (0.1% DMSO); FF, fenofibrate.

We next studied whether fenofibrate inhibits NF-κB activation. Because NF-κB is activated through phosphorylation-dependent ubiquitination and proteasome-mediated degradation of IκBα [26], we examined the inhibitory effect of fenofibrate on IκBα phosphorylation and degradation. As shown in figure 3A, LPS-induced phosphorylation and degradation of IκBα was significantly decreased in fenofibrate-treated BMDMs from WT mice. In contrast, in BMDMs from SHP KO mice, higher activation of NF-κB signaling was observed in control and fenofibrate-treated mice (Fig. 3A). The nuclear translocation of NF-κB p65 was similarly inhibited in fenofibrate-treated WT cells, but not in SHP-KO cells (Fig. 3B), suggesting that NF-κB signaling inhibition mediated by fenofibrate is dependent on SHP. We previously showed that SHP-mediated NF-κB signaling inhibition requires SHP interaction with the NF-κB subunit p65 in a resting status, which shifted to an association with the adaptor TRAF6 after TLR signaling [18]. Interestingly, fenofibrate resulted in a strong interaction between SHP and the NF-κB subunit p65, which was reduced by LPS stimulation (Fig. 3C). Moreover, LPS-induced polyubiquitination of TRAF6 was inhibited by fenofibrate treatment in WT BMDMs; however, this was more elevated with or without fenofibrate treatment, in SHP-knockdown BMDMs (Fig. 3D). Taken together, these results indicate that fenofibrate inhibits LPS-mediated inflammatory signaling activation through SHP.

### Fenofibrate Regulates TLR-dependent Inflammatory Signaling in a PPARα-independent Manner

**Figure 4:**
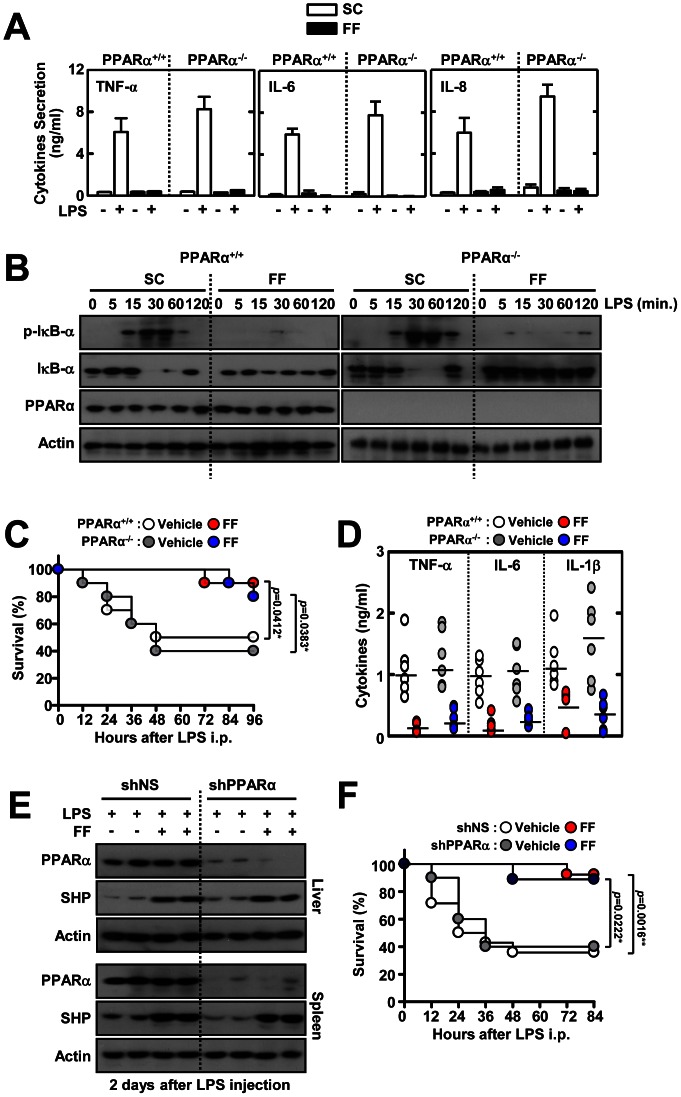
PPARα is not involved in fenofibrate-mediated regulation of inflammatory responses *in vitro* and *in vivo*. (**A** and **B**) BMDMs from *Pparα* ^+/+^ and *Pparα* ^−/−^ mice were treated with fenofibrate (50 µM) for 4 h, and then stimulated with LPS (100 ng/mL) for 18 h (**A**) or the indicated times (**B**). (**A**) ELISA analyses of TNF-α, IL-6, and IL-8 proteins in culture supernatants. (**B**) Immunoblot (IB) analyses of phosphorylated and total forms of IκB-α and PPARα. Actin was used as a loading control. The data are representative of at least three independent experiments with similar results. (**C** and **D**) *Pparα* ^+/+^ and *Pparα* ^−/−^ mice received fenofibrate (100 mg/kg; administrated orally) or vehicle for 7 consecutive days, followed by injection with LPS (30 mg/kg; n = 10 mice per group, i.p) for the indicated periods (**C**) or 18 h (**D**). (**C**) The survival of *Pparα* ^+/+^ and *Pparα* ^−/−^ mice was monitored for 96 h. Statistical differences (*), as compared to control mice, are indicated (log-rank test). (**D**) Sera were collected from *Pparα* ^+/+^ and *Pparα* ^−/−^ mice (n = 5 each group) at 18 h post-LPS injection and the concentrations of TNF-α, IL-6, and IL-1β were measured by ELISA. (**E** and **F**) Mice were injected with lentivirus (1×10^9^ pfu; administered intravenously) expressing nonspecific shRNA (shNS) or shRNA specific for PPARα (shPPARα) with polybrene (8 µg/mL) for 2 consecutive days and then orally administered fenofibrate for 7 consecutive days before LPS challenge (30 mg/mL, i.p). Mortality was measured for 10 mice per group for 84 h. (**E**) Protein expression of PPARα and actin in liver and spleen was analyzed by IB 2 days after LPS injection. (**F**) The survival of shNS- and shPPARα-injected mice was monitored for 84 h. Statistical differences, as compared to the control mice, are indicated (log-rank test). The data (**B** and **E**) are representative of at least three independent experiments with similar results. Quantitative data are shown as the mean ± SD of three experiments (**A** and **D**). FF, fenofibrate.

Because fenofibrate is a well-known PPARα agonist, we determined the effects of fenofibrate in regulating cytokine production in BMDMs from WT and PPARα KO mice. It was noted that LPS-induced proinflammatory cytokine production was substantially increased in PPARα KO cells, as compared to WT cells (Fig. 4A), indicating an anti-inflammatory role for PPARα, as suggested in previous studies [27]. However, fenofibrate dramatically reduced the secretion of proinflammatory cytokines (TNF-α, IL-6, and IL-8) in both BMDMs from WT and PPARα KO mice (Fig. 4A). Additionally, fenofibrate treatment led to a significant decrease in LPS-induced phosphorylation and degradation of IκBα in WT and PPARα-KO BMDMs (Fig. 4B). In addition, LPS-induced phosphorylation and degradation of IκBα was considerably increased in PPARα-deficient BMDMs, as compared to WT cells (Fig. 4B), suggesting a negative regulatory role for PPARα in NFκB-signaling.

We next assayed the *in vivo* role of PPARα in fenofibrate-mediated regulation of systemic inflammation. When WT and PPARα KO mice were succumbed to LPS challenge, there was no significant difference in survival rates between WT and PPARα KO mice after treatment with or without fenofibrate (Fig. 4C). Similar to survival rates, both groups produced markedly decreased levels of proinflammatory cytokines (TNF-α, IL-6, and IL-1β) when fenofibrate was administered before LPS challenge (Fig. 4D). We further examined the effects of *in vivo* PPARα silencing on the susceptibility against septic shock. The lentiviral particles carrying shRNA against PPARα (shPPARα) were intravenously injected into C57BL/6 WT mice. After sacrifice 2 or 7 days post-injection, the liver and spleen were dissected, homogenized, and protein extracts were analyzed by western blotting to determine PPARα levels. In the mice injected with shPPARα for 7 days, PPARα was markedly reduced in the liver and spleen (Fig. 4E). However, fenofibrate-induced SHP expression was not different in liver and spleen tissues from either group (Fig. 4E). When we subjected control shRNA lentivirus (shNS)- and shPPARα-injected mice to septic shock, there was no statistical significance in overall survival rate between shNS- and shPPARα-injected (Fig. 4F). Fenofibrate administration significantly inhibited the mortality in both shNS- and shPPARα-injected groups (Fig. 4F). Taken together, these results demonstrate that PPARα is not associated with fenofibrate-mediated anti-inflammatory responses *in vivo*.

### Fenofibrate Induces SHP through a Serine/threonine Kinase 11 (LKB1)-AMP Activated Protein Kinase (AMPK) Pathway

**Figure 5:**
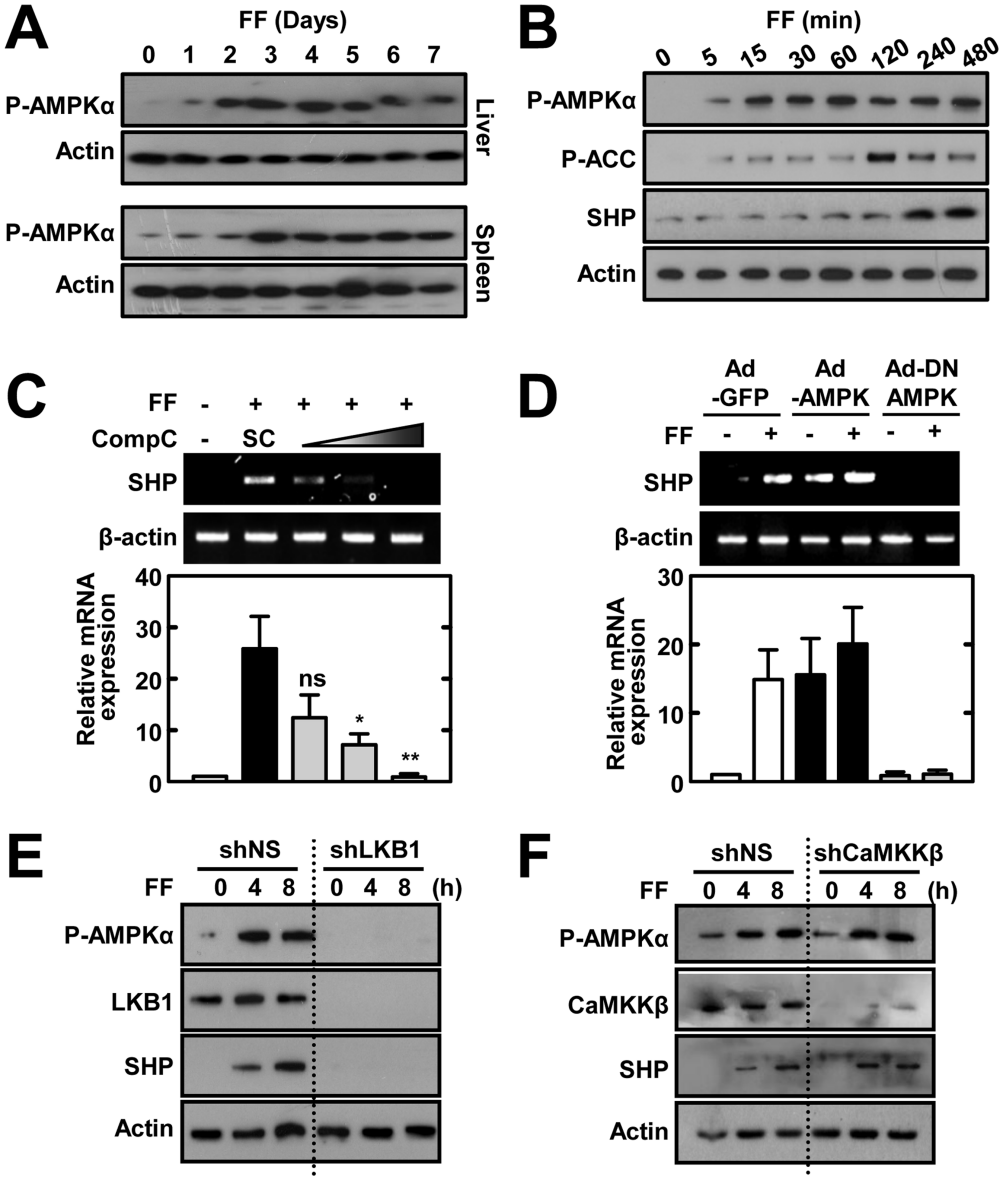
Fenofibrate induces SHP expression through LKB1-dependent AMPK signaling. (**A**) WT mice (*n* = 3) received fenofibrate (100 mg/kg; administrated orally) or vehicle only for the indicated period. The expression of phosphorylated AMPKα in liver and spleen was assessed by immunoblotting (IB). Whole cell lysates (WCL) were used for IB with anti-Actin. (**B**) BMDMs were treated with fenofibrate (50 µM) for various times, followed by IB with phosphorylated forms of AMPKα, ACC, and SHP. WCL were used for IB with anti-β-actin as the loading control. (**C**) BMDMs were treated with fenofibrate (50 µM for 6 h) in the presence or absence of the AMPK inhibitor compound C (Comp C; 5, 10, or 25 µM). Total RNA was extracted from WCL and used for RT-PCR analyses of *Shp* and *Actb* mRNA; below, densitometry. (**D**) BMDMs were transduced for 48 h with adenovirus encoding GFP only (Ad-GFP), constitutively active AMPK (Ad-AMPK), or dominant negative AMPK (Ad-DN-AMPK; MOI = 10) and were then treated with fenofibrate (50 µM) for 4 h. Total RNA was extracted from WCL and analyzed by semi-quantitative RT-PCR for *Shp* and *Actb* mRNA. Representative gel images (*top*); densitometric analyses (*bottom*). (**E** and **F**) BMDMs were transduced with shNS or shLKB1 (E) or shCAMKKβ (F) prior to treatment with fenofibrate (50 µM) for the indicated time periods, followed by IB with antibodies against the phosphorylated forms of AMPKα and SHP. WCL were used for IB with anti-β-actin as the loading control. Total LKB1 (D) and CAMKKβ (E) protein expression was measured for transduction efficiency of lentiviral vectors. The data are representative of at least three independent experiments with similar results. Quantitative data are shown as the mean ± SD of three experiments (**C** and **D** *bottom*). Statistical differences (*, *p*<0.05; **, *p*<0.01), as compared to the control cultures, are indicated (paired *t*-test with Bonferroni adjustment). FF, fenofibrate. *n.s.*, non-specific.

To evaluate the upstream signaling pathways of SHP gene and protein expression, we examined the phosphorylation of AMPK, a key signaling step involved in SHP expression. Treatment of BMDMs with fenofibrate considerably increased the *in vivo* (Fig. 5A; liver and spleens) and *in vitro* (Fig. 5B; BMDMs) phosphorylation of the Thr^172^ residue in AMPK, which is located in the critical activation loop AMPK α subunit [28]. In addition, the phosphorylation of acetyl CoA-carboxylase at Ser^79^, the best known downstream target of AMPK [29], followed after AMPK phosphorylation in BMDMs after fenofibrate treatment (Fig. 5B). Pretreatment of BMDMs with Compound C, a potent ATP-competitive inhibitor of AMPK activity, or transduction with adenovirus containing the dominant negative form of AMPK (Ad-DN-AMPK), significantly blocked SHP gene expression (Fig. 5C and 5D). We further investigated the role of two known upsteam kinases, LKB1 (Peutz-Jerhers protein) and Ca^2+^/calmodulin-dependent protein kinase kinase β (CaMKKβ) [28], in the activation of fenofibrate-induced AMPK pathway and SHP expression. As shown in figure 5E and 5F, fenofibrate failed to induce AMPK phosphorylation and SHP expression in the absence of LKB1, but not by inhibition of CaMKK. These results indicate that fenofibrate action on SHP expression in BMDMs is dependent on LKB1-AMPK activation pathways.

### Fenofibrate Suppresses LPS-induced Mitochondrial ROS Generation and Pro-inflammatory Responses via SHP-induced UCP2 Expression

**Figure 6:**
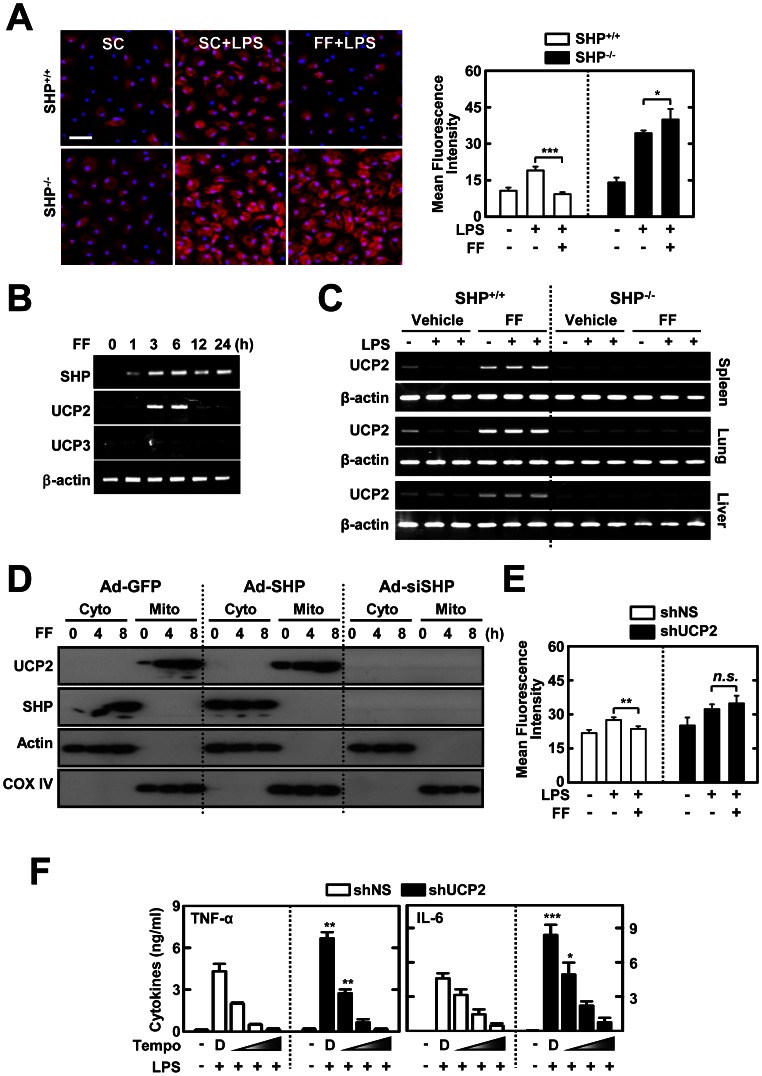
Fenofibrate-induced SHP inhibits the generation of mitochondrial ROS and pro-inflammatory cytokines through UCP2 expression. (**A**) BMDMs from *Shp* ^+/+^ and *Shp* ^−/−^ mice were pretreated with solvent control (SC) or fenofibrate (FF; 50 µM) for 4 h, and then stimulated with LPS (100 ng/mL) for 15 min. Cells were labeled with MitoSOX for 30 min and analyzed for mitochondrial ROS (red) using immunofluorescence microscopy; nuclei are stained with the DAPI (blue). Scale bar, 20 µm. *Right*, quantification of fluorescence intensity. (**B**) BMDMs were pretreated with fenofibrate (50 µM) for various times and analyzed using semi-quantitative RT-PCR for *Shp*, *Ucp2*, *Ucp3*, and *Actb* mRNA. (**C**) *Shp* ^+/+^ and *Shp* ^−/−^ mice received fenofibrate (100 mg/kg; administrated orally) or vehicle only for 7 consecutive day before LPS injection (30 mg/kg; *n* = 15 each group, i.p). Six hours after the LPS challenge, mice were sacrificed and the mRNA expression of *Ucp2* and β-actin was assessed by semi-quantitative RT-PCR analyses in liver, spleen, or lung tissues. (**D**) BMDMs were transduced with Ad-GFP, Ad-SHP, or Ad-siSHP (MOI = 10) for 48 h and then treated with fenofibrate (50 µM) for the indicated time periods. The cells were subcellularly fractionated and subjected to IB analyses using anti-UCP2 and anti-SHP. Actin and COX IV are cytoplasmic and mitochondrial markers, respectively. (**E** and **F**) BMDMs transduced with lentivirus expressing nonspecific shRNA (shNS) or shRNA specific for UCP2 (shUCP2) were treated with fenofibrate (50 µM; 4 h), followed by LPS (100 ng/ml; 15 min) stimulation. For F, BMDMs were pretreated with mitochondrial ROS scavengers (mitoTEMPO; 10, 50, 100 µM). (**E**) Quantitative analyses of mitochondrial ROS, measured by staining with MitoSOX, as described in Figure 6A. (**F**) ELISA analyses of TNF-α and IL-6 in culture supernatants. The data (**A** left, **B**, **C**, and **D**) are representative of at least three independent experiments with similar results. Quantitative data are shown as the mean ± SD of three experiments (**A** right, **E**, and **F**). Statistical differences (*, *p*<0.05; **, *p*<0.01; ***, *p*<0.001) are indicated (paired *t*-test with Bonferroni adjustment). FF, fenofibrate.

Given that the hypolipidemic drug fenofibrate can reduce the generation of ROS [30] and that LPS-induced inflammatory cytokine production is linked to ROS from mitochondrial origin [31], we hypothesized that fenofibrate-induced SHP inhibits inflammatory cytokine production by modulating mitochondrial ROS generation. SHP deficiency resulted in higher mitochondrial ROS generation after LPS stimulation, as compared to control conditions (Fig. 6A). Fenofibrate markedly abrogated the production of mitochondrial ROS in WT-BMDMs after LPS stimulation. However, mitochondrial ROS was greatly elevated and not decreased by fenofibrate treatment in SHP-deficient cells (Fig. 6A).

Previously, it was shown that the mitochondrial anion carrier protein UCP2 is crucial for modulating mitochondrial ROS and inflammatory responses [19], [21]. We hypothesized that SHP participates in regulating UCP2 expression to modulate mitochondrial ROS production. In response to fenofibrate treatment, gene expression of UCP2, but not UCP3, was significantly induced in BMDMs (Fig. 6B) and *in vivo* (Fig. 6C). Subcellular fractionation followed by western blot showed that UCP2 protein was detected in the mitochondrial fraction of macrophages after fenofibrate treatment, whereas SHP was observed in the cytosolic fraction (Fig. 6D). Importantly, fenofibrate-induced UCP2 expression in the mitochondrial compartment was dependent on SHP expression, because it was greatly attenuated in SHP-knockdown cells (Fig. 6D), suggesting that SHP is required for UCP2 expression. Silencing of UCP2 in BMDMs led to increased mitochondrial ROS generation after LPS stimulation (Fig. 6E). Fenofibrate treatment markedly inhibited the LPS-induced mitochondrial ROS generation in shNS-transduced BMDMs, whereas it did not inhibit LPS-induced generation of mitochondrial ROS in shUCP2-transduced BMDMs (Fig. 6E). Furthermore, treatment of the mitochondria-targeted antioxidant mitoTEMPO dose-dependently attenuated production of proinflammatory cytokines (TNF-α and IL-6) in shUCP2-transduced BMDMs (Fig. 6F), suggesting that enhanced mitochondrial ROS production by UCP2 knockdown promotes proinflammatory responses in these cells. Overall, these data show that SHP-induced UCP2 is involved in fenofibrate-mediated inhibition of mitochondrial ROS generation, which contributes to proinflammatory responses in macrophages after LPS stimulation.

### UCP2 is Involved in Fenofibrate-mediated Anti-inflammatory Responses during Endotoxin-induced Septic Shock

**Figure 7:**
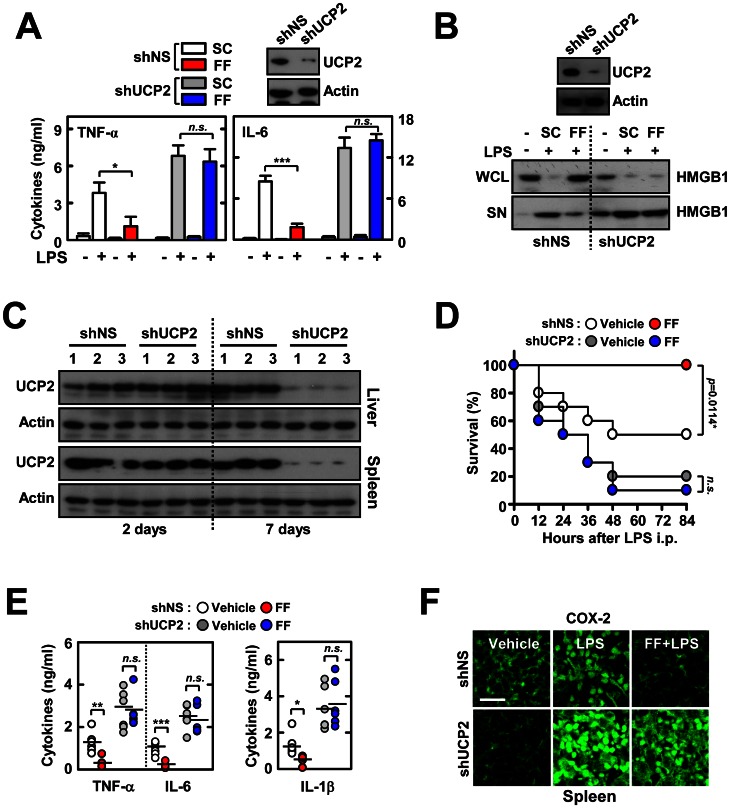
UCP2 is required for the fenofibrate-mediated protective activity against LPS-induced lethal shock. (**A**) BMDMs were transduced with lentivirus expressing nonspecific shRNA (shNS) or shRNA specific for UCP2 (shUCP2), followed by treatment with fenofibrate (50 µM; 4 h) and stimulation with LPS (100 ng/ml; 18 h). The supernatants were collected and protein expression of TNF-α, IL-6, and IL-8 was determined using ELISA. (**B**) Immunoblot analysis of HMGB1 protein in whole cell lysate (WCL) and supernatant (SN). (**C** to **F**) Mice were injected with lentivirus (1×10^9^ pfu; administered intravenously) expressing nonspecific shRNA (shNS) or shRNA specific for UCP2 (shUCP2) with polybrene (8 µg/mL) for 2 consecutive days and then orally administered fenofibrate for 7 consecutive days before LPS challenge (30 mg/mL, i.p). (**C**) Expression of UCP2 in liver and spleen at 2 and 7 days after i.v. infection with lentivirus was assessed by immunoblotting (IB). Whole cell lysates (WCL) were used for IB with anti-Actin. (**D**) The survival of mice (*n* = 10 mice per group) was monitored for 84 h. Results are presented as the mean ± SEM. *n.s.*, non-specific (log-rank test). (**E**) Sera were collected from each group (n = 5) at 18 h after i.p LPS injection and the concentrations of TNF-α, IL-6, and IL-1β were measured using ELISA. (**F**) Representative immunofluorescence images for expression of COX-2 in spleen tissues from each group. Scale bar, 50 µm. The data (**B**, **C**, and **F**) are representative of at least three independent experiments with similar results. Quantitative data are shown as the mean ± SD of three experiments (**A** and **E**). Statistical differences (**, *p*<0.01; ***, *p*<0.001) are indicated (paired *t*-test with Bonferroni adjustment). FF, fenofibrate.

It was previously shown that disruption of *Ucp2* in mice results in constitutively activated NF-κB signaling and amplifies inflammatory responses *in vivo* and *in vitro* [32]. Therefore, we examined whether SHP-induced UCP2 is required for fenofibrate-induced negative regulation of inflammatory responses to LPS. As shown in figure 7A, UCP2 knockdown by specific lentiviral transduction led to an increase in LPS-induced proinflammatory cytokine secretion in BMDMs. However, fenofibrate-mediated inhibition of cytokine production (Fig. 7A) and HMGB1 release (Fig. 7B) was not observed in UCP2-knockdown cells.

To determine whether UCP2 is involved in the fenofibrate-induced protection against endotoxin-induced lethal shock *in vivo*, we intravenously injected lentiviral particles carrying shRNA against UCP2 (shUCP2) into WT mice before challenging with LPS. After sacrifice 7 days post-injection, UCP2 levels were considerably reduced in liver and spleen (Fig. 7C). We determined the effects of *in vivo* silencing of UCP2 on fenofibrate-induced regulation of mortality and inflammatory mediator generation against septic shock. As shown in figure 7D, *in vivo* reduced expression of UCP2 resulted in significantly lower survival rates, as compared to the shNS-injected mice group. Administration of fenofibrate in the shNS-injected control group significantly improved the survival rates after LPS challenge, whereas the same treatment did not recover mice survival when subjected to septic shock (Fig. 7D). Consistent with survival rates, the sera levels of proinflammatory cytokine production (Fig. 7E) were significantly higher in the shUCP2-injected group after LPS challenge. Fenofibrate treatment markedly decreased serum inflammatory cytokine levels in shNS-injected mice subjected to lethal shock. However, this was not observed in the shUCP2-injected group (Fig. 7E). Moreover, the expression of cyclooxygenase-2 was markedly attenuated in spleens from LPS-challenged WT mice upon treatment with fenofibrate, whereas this was not observed in UCP2-knockdown mice (Fig. 7F). Taken together, these results demonstrate that UCP2 is critically involved in fenofibrate-mediated anti-inflammatory responses during endotoxin-induced septic shock.

## Discussion

SHP negatively regulates inflammation [18] but it remained unresolved whether inducing agents of SHP could modulate the systemic inflammation. In this study, we provide evidence that the anti-lipidemic drug fenofibrate significantly induces SHP *in vivo* and *in vitro*, and inhibits systemic inflammatory responses through SHP. Importantly, fenofibrate-induced SHP expression was required for UCP2, a mitochondrial uncoupler, *in vivo* and *in vitro*. Our study also showed that lack of either SHP or UCP2 results in a ‘primed’ state of inflammatory responses and, thus, upregulates pro-inflammatory cytokine production through increased mitochondrial ROS generation.

Because fenofibrate is a well-known ligand for PPARα, an important nuclear receptor and transcription factor that plays a key role in controlling lipid metabolism and inflammation [33], it is remarkable that fenofibrate modulates inflammatory responses and endotoxin-induced lethal shock, independently of PPARα. Fenofibrate is widely used in clinical practice as a lipid-lowering drug, and is useful for the treatment of hypercholesterolemia, mixed dyslipidemia, and hypertriglyceridemia [34]. Recent animal and clinical studies have proposed a potential use of fenofibrate in the treatment of several inflammatory diseases, including microvascular complications [35], diabetic retinopathy [36], Japanese encephalitis viral infection [37], and rheumatoid arthritis with dyslipidemia [38]. Previous reports using PPARα-deficient mice showed that several of the beneficial effects of fenofibrate on the relief of ischemia reperfusion injury and acute-phase inflammatory responses are mediated through a PPARα-dependent mechanism [39], [40]. It was also thought that the main lipid-modulation activity mediated by fenofibrate is PPARα-dependent [34], [35], [41]. However, the mechanism by which fenofibrate modulates a variety of non-lipid functions has not been completely understood.

Previous studies demonstrated that IL-1- or IL-6-mediated acute-phase responses are regulated by fenofibrate in liver-specific PPARα-expressing mice [39]. Recently, it was reported that fenofibrate requires the PPARα gene to improve dyslipidemia and liver steatosis in mice after they were fed a western diet. However, atherogenesis does not require PPARα [42]. Our findings partly correlate with previous data showing that fenofibrate operates a PPARα-independent AMPK-SHP regulatory cascade, ameliorates hepatic metabolic syndromes, and inhibits gene induction of plasminogen activator inhibitor type I [11]. Together, these data indicate that fenofibrate action in pleiotropic functional regulation is mediated through a PPARα-dependent or independent manner, and depends on the tissue distribution of specific receptors, different cell types, and the route of administration of fenofibrate.

The current data are consistent with previous studies by Chanda et al. [11], in which fenofibrate increased SHP expression in liver cells and mouse liver via AMPK signaling. AMPK is a serine/threonine kinase that regulates glucose and lipid metabolism, thus maintaining energy homeostasis [43]. The impaired control of AMPK activity can lead to insulin resistance and metabolic syndromes [43]. However, emerging data also indicate that AMPK signaling negatively modulates inflammatory responses by inhibiting NF-κB signaling [44]. Indeed, numerous studies have demonstrated that AMPK represses inflammatory responses induced by different stimuli [45]–[49]. Several AMPK-activating agents are known to modulate inflammatory responses. For example, metformin, the first-line medication for type 2 diabetes, exhibits anti-inflammatory activities in macrophages and systemic inflammation *in vivo* by decreasing the level of proinflammatory mediators [50], [51]. The present data show that fenofibrate mediates anti-inflammatory responses by interacting with NF-κB p65, which correlates with data from our previous studies [18]. AMPK is activated by an increased AMP/ATP ratio as well as by phosphorylation of the α subunit (Thr^172^) via the upstream kinases LKB1, CaMKKβ, and transforming growth factor-β-activated kinase 1 [43], [44]. Although it is known that fenofibrate inhibits lipid accumulation and microvascular inflammatory responses through AMPK activation [52], [53], the upstream or downstream signaling mechanisms by which fenofibrate induced anti-inflammatory responses remain to be characterized. The current data also show that in macrophages, fenofibrate-induced AMPK activation is regulated by LKB1, but not by CaMKKβ. In support of these findings, recent data indicate that MSP-induced SHP expression is regulated by LKB1, but not Ca^2+^-CaMKK pathways, in macrophages [18].

Our findings reveal a previously unknown mechanism of fenofibrate-induced anti-inflammation through an AMPK-SHP-UCP2 pathway. Recent studies have indicated that mitochondrial ROS, acting as signaling molecules, are responsible for triggering pro-inflammatory responses [31], [54], [55]. However, a role for mitochondrial ROS in SHP-deficient cells in amplifying proinflammatory responses has not been demonstrated. Our data reveal that SHP is required for UCP2 expression and regulation of mitochondrial ROS generation. Increased mitochondrial ROS are found in diverse chronic inflammatory diseases such as neurodegeneration, Crohn's disease, and cancer [56]. Mitochondrial UCP2 is implicated in various physiological and pathological processes through homeostatic regulation of mitochondrial ROS production [20], [57]. Our data partly correlate with previous findings showing that UCP2 plays an essential role inhibiting mitochondrial ROS generation and macrophage inflammatory responses [1]. Moreover, increased mitochondrial ROS generation by silencing *Ucp2* was found to be involved in enhancing inflammatory responses in these cells in response to LPS. These data also correlate with previous studies demonstrating that *Ucp2*-deficient cells have elevated NF-κB signaling activation, which potentiates the responses of inflammatory cytokines [31].

SHP is recognized as a nuclear transcriptional repressor [4]; however, it is found in the cytosol of immune cells as a key negative regulator in TLR-mediated inflammation via its association with signaling molecules such as TRAF6 [18]. Previous studies have also shown that SHP targets to the mitochondria and mediates mitochondrial function and apoptosis in tumor cells [58]. In the present study, we found that fenofibrate efficiently induces SHP, which negatively regulates inflammation through two potential phases: one through direct inhibition of NF-κB signaling by interacting with NF-κB p65 in the cytosolic compartments, and the other by induction of UCP2 gene expression, which is responsible for modulation of mitochondrial ROS generation and inflammatory cytokine production. Together, these data show distinct molecular mechanisms by which fenofibrate treatment inhibits systemic inflammatory responses through SHP by regulating NF-κB signaling and inducing UCP2, thus down-regulating ROS generation from the mitochondria.
